# Large Language Model–Based Chatbots and Agentic AI for Mental Health Counseling: Systematic Review of Methodologies, Evaluation Frameworks, and Ethical Safeguards

**DOI:** 10.2196/80348

**Published:** 2026-03-13

**Authors:** Ha Na Cho, Jiayuan Wang, Di Hu, Kai Zheng

**Affiliations:** 1 Department of Informatics University of California, Irvine Irvine, CA United States

**Keywords:** conversational agent, digital health, digital mental health intervention, large language model chatbots, personalized health care

## Abstract

**Background:**

Large language model (LLM)–based chatbots have rapidly emerged as tools for digital mental health (MH) counseling. However, evidence on their methodological quality, evaluation rigor, and ethical safeguards remains fragmented, limiting interpretation of clinical readiness and deployment safety.

**Objective:**

This systematic review aimed to synthesize the methodologies, evaluation practices, and ethical or governance frameworks of LLM-based chatbots developed for MH counseling and to identify gaps affecting validity, reproducibility, and translation.

**Methods:**

We searched Google Scholar, PubMed, IEEE Xplore, and ACM Digital Library for studies published between January 2020 and May 2025. Eligible studies reported original development or empirical evaluation of LLM-driven MH counseling chatbots. We excluded studies that did not involve LLM-based conversational agents, were not focused on counseling or supportive MH communication, or lacked evaluable system outputs or outcomes. Screening and data extraction were conducted in Covidence (Veritas Health Innovation) following PRISMA (Preferred Reporting Items for Systematic Reviews and Meta-Analyses) 2020 guidelines. Study quality was appraised using a structured traffic light framework across 5 methodological domains (design, dataset reporting, evaluation metrics, external validation, and ethics), with an overall judgment derived across domains. We used narrative synthesis with descriptive aggregation to summarize methodological trends, evaluation metrics, and governance considerations.

**Results:**

Twenty studies met the inclusion criteria. GPT-based models (GPT-2/3/4) were used in 45% (9/20) of studies, while 90% (18/20) used fine-tuned or domain-adaptation models such as LLaMa, ChatGLM, or Qwen. Reported deployment types were not mutually exclusive; standalone apps were most common (18/20, 90%), and some systems were also implemented as virtual agents (4/20, 20%) or delivered via existing platforms (2/20, 10%). Evaluation approaches were frequently mixed, with qualitative assessment (13/20, 65%), such as thematic analysis or rubric-based scoring, often complemented by quantitative language metrics (18/20, 90%), including BLEU (Bilingual Evaluation Understudy), ROUGE (Recall-Oriented Understudy for Gisting Evaluation), or perplexity. Quality appraisal indicated consistently low risk for dataset reporting and evaluation metrics, but recurring limitations were observed in external validation and reporting on ethics and safety, including incomplete documentation of safety safeguards and governance practices. No included study reported registered randomized controlled trials or independent clinical validation in real-world care settings.

**Conclusions:**

LLM-based MH counseling chatbots show promise for scalable and personalized support, but current evidence is limited by heterogeneous study designs, minimal external validation, and inconsistent reporting of safety and governance practices. Future work should prioritize clinically grounded evaluation frameworks, transparent reporting of model and prompt configurations, and stronger validation using standardized outcomes to support safe, reliable, and regulatory-ready deployment.

## Introduction

The increasing global burden of mental health (MH) disorders necessitates accessible and scalable intervention strategies [1]. Persistent challenges, such as provider shortages, access disparities, and delayed care, continue to limit timely support for individuals in need. In this context, digital mental health interventions (DMHIs) have emerged as potential tools to complement existing services, particularly for supportive counseling and early intervention [2]. Among these, large language model (LLM)–based chatbots have emerged as effective tools for delivering scalable psychological counseling and intervention strategies. These artificial intelligence (AI)–driven systems can respond instantly, adapt to user needs, and offer personalized support, making them a valuable resource for individuals who lack immediate access to human experts. However, despite the growing interest in the DMHI domain, concerns and challenges remain regarding their effectiveness and reliability in clinical practice. Addressing these limitations requires a comprehensive assessment of current LLM-based chatbot methodologies, their evaluation frameworks, and their role in MH counseling.

Previous systematic reviews have explored the effectiveness of rule-based and traditional AI-driven chatbots for DMHI. Early studies primarily focused on text-based conversational agents that followed prescripted dialogues or used traditional machine learning models trained on structured datasets [3,4]. These reviews highlight the potential of AI-based DMHI to enhance user engagement and symptom management, while also exposing key limitations such as rigid responses, limited adaptability, and a lack of contextual understanding [5,6]. More recent reviews have examined LLMs’ integration in MH, demonstrating that LLM-based chatbots exhibit superior natural language understanding, response flexibility, and contextual understanding compared to earlier MH chatbot architectures [7-9]. However, many of these reviews adopt a broad focus, assessing LLM applications in MH for education, screening, and assessment, rather than examining their role in counseling-specific interventions [10,11]. This gap leaves uncertainty regarding the effectiveness and clinical applicability of LLMs explicitly designed for MH counseling. Despite the increasing adoption of LLMs in digital therapy, existing studies have yet to systematically assess their clinical effectiveness, response accuracy, and real-world impact in MH counseling scenarios [12]. Despite their promise, LLM-based counseling systems raise substantial ethical and safety concerns, including biased responses, hallucinated information, inappropriate reassurance, and potential harm when interacting with emotionally vulnerable users. These risks motivate the need for systematic evaluation of both methodological rigor and ethical safeguards.

Recent advances in MH-focused LLMs demonstrated substantial improvements in natural language processing (NLP), contextual awareness, and response generation. Transformer-based models such as GPT series, LLaMa, MedAlpaca [13], and ChatDoctor [14] have been increasingly integrated for MH applications, offering real-time user interaction and context-aware counseling support. These models can analyze user queries, infer emotional states, and generate human-like responses, providing automated counseling services to users. Moreover, fine-tuned LLMs tailored to MH applications, notably MentalLlama [15], Mistral [16], and MentalBART [17], have shown promise in outperforming general models on key counseling metrics. Despite these advancements, existing models still face significant challenges, including biases in training data, potential misinformation, lack of clinical validation, and ethical concerns related to overreliance on AI-generated counseling [18]. Furthermore, rigorous evaluation frameworks to ensure clinical efficacy, safety, and real-world applicability remain limited, highlighting the need for standardized assessment methodologies in MH-focused LLM deployment [19].

While recent reviews have LLMs in MH broadly, most adopt a wide scope encompassing screening, education, and assessment tasks. In contrast, this review focuses specifically on LLM-based systems designed for counseling and supportive dialogue, where conversational appropriateness, safety, and ethical governance are central concerns. We systematically examine how these counseling-oriented systems are developed and evaluated, including model preprocessing, prompt engineering, fine-tuning strategies, deployment formats, and reported safeguards against harm. By synthesizing methodological, evaluative, and ethical patterns across studies, this review provides a focused analysis of the current landscape of LLM-driven MH counseling and identifies key gaps relevant to future research and clinical integration. Accordingly, this review was guided by three primary questions:

1. How have LLMs been applied and evaluated for MH counseling or support tasks?
2. What types of clinical or psychometric outcomes have been measured to assess their effectiveness and safety?
3. How have studies addressed ethical considerations such as bias, misinformation, and user safety when deploying LLM-based chatbots or agentic systems?

## Methods

### Search Strategy

This study followed PRISMA (Preferred Reporting Items for Systematic Reviews and Meta-Analyses) guidelines, ensuring a structured and transparent review process. We conducted a systematic literature search across Google Scholar, PubMed, IEEE Xplore, and ACM Digital Library, covering studies published between January 2020 and May 2025. The search queries incorporated LLM-related terms (“large language model” OR “LLM” OR “LLMs”) and MH-specific terms (“mental illness” OR “mental disorder” OR “mental health” OR “mental wellness”), along with intervention-focused keywords (“chatbot” OR “counseling” OR “conversational” OR “digital intervention” or “DMHI”). The final search queries were iteratively refined using Boolean logic and expert input and were performed on May 15, 2025, and study screening and data extraction were completed on June 10, 2025. The full database-specific search queries are provided in Multimedia Appendix 1 and operational definitions of key technical terms used for coding and synthesis are provided in Multimedia Appendix 2. The PRISMA checklist is provided in Multimedia Appendix 3.

Study screening, data extraction, and methodological evaluation were conducted collaboratively by 3 reviewers (HC, JW, and DH) using Covidence (Veritas Health Innovation) and Zotero (Corporation for Digital Scholarship, CDS). The reviewers independently screened titles, abstracts, and full texts to determine study eligibility based on predefined inclusion and exclusion criteria. Discrepancies in study selection and data extraction were discussed during weekly meetings, where consensus was reached through deliberation. Any disagreements that persisted were resolved through majority voting, ensuring consistency and rigor in the systematic review process.

**Figure 1:**
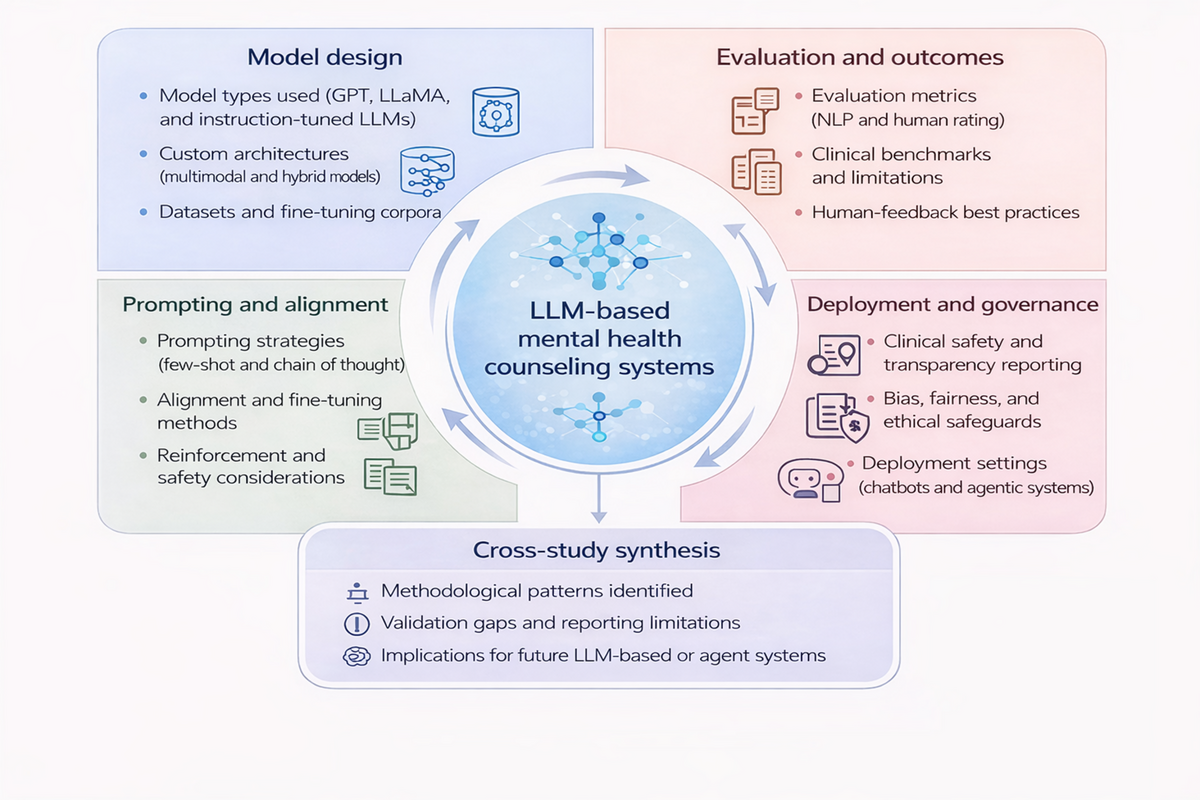
Conceptual framework of the systematic review. LLM: large language model; NLP: natural language processing.

The initial search retrieved 1593 studies, which underwent a multistage screening process, including duplication removal, title and abstract review screening, followed by full-text analysis. After applying inclusion and exclusion criteria, a total of 20 studies [20-39] were included in the final review. Figure 1 presents the conceptual framework of this systematic review, illustrating how extracted methodological evidence is synthesized and translated into downstream design and evaluation considerations for LLM-based MH systems.

### Inclusion and Exclusion Criteria

Studies were included if they evaluated LLM-based chatbots for MH counseling and reported performance measures on response quality, engagement, effectiveness, or ethical considerations, with only English-language studies considered. Only original peer-reviewed studies published between 2020 and 2025 were considered. Studies implementing fine-tuning, prompt engineering, or structured evaluation methodologies were included for comparative analysis. Studies were excluded if a study design misaligned with the research objectives, focused on rule-based chatbots, assessed general-purpose conversational AI without an MH focus, or lacked empirical evaluation. Review articles, opinion pieces, and non–peer-reviewed sources were also excluded. In addition, preprints and conference proceedings were screened when they reported original empirical evaluations of LLM-based MH counseling systems and provided sufficient methodological detail to permit appraisal. Preprints were included selectively to capture emerging methodological approaches in this rapidly evolving field; however, non–peer-reviewed records with insufficient methodological detail were excluded, and preprints were interpreted with appropriate caution alongside peer-reviewed evidence.

### Data Collection and Extraction

This review extracted key model attributes, development methodologies, evaluation frameworks, and study outcomes across the studies on LLM-based MH chatbots. Extracted data fields included LLM and version (eg, GPT-4, LLaMA, and ChatGLM2), given model name, and service approach (eg, standalone chatbot, integrated platform, mobile app, or virtual LLM agent). Additionally, we gathered details on dataset characteristics, distinguishing between studies that used publicly available datasets, proprietary datasets, or synthetically generated datasets for fine-tuning. Preprocessing techniques were identified, including text normalization, augmentation, and filtering. For model development, we extracted information on prompting strategies, as well as fine-tuning methods. Evaluation methodologies were categorized into qualitative and quantitative approaches. We identified human evaluation metrics, such as expert-reviewed response accuracy, empathy, and coherence, along with automated quantitative evaluation methods (eg, BLEU [Bilingual Evaluation Understudy] and ROUGE [Recall-Oriented Understudy for Gisting Evaluation]). Furthermore, the review examined whether LLM-generated responses were fully automated, human-in-the-loop, or used for backend knowledge retrieval. Notably, not all studies provided information across all extracted fields, and when data were unavailable, it was marked as “NA.”

Given the technical focus of this review, outcome measures were extracted primarily in relation to model performance and evaluation methodology rather than user-reported or clinical outcomes. Where available, outcomes were categorized as user-reported or expert-assessed for clarity, though most studies emphasized model-level performance metrics.

### Risk of Bias and Study Quality

**Figure 2:**
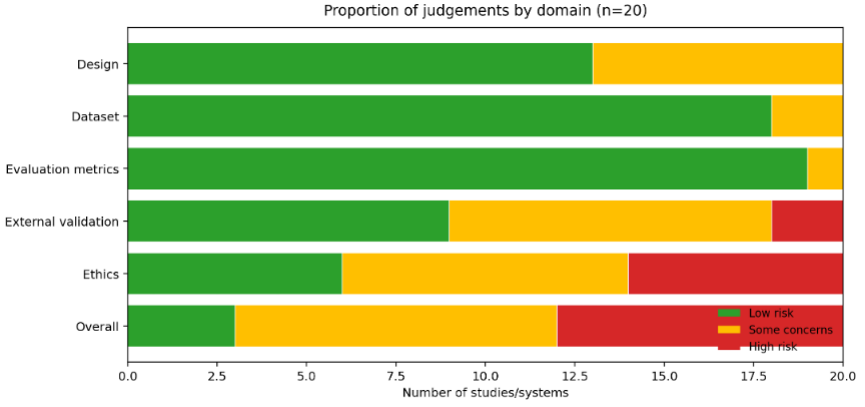
Summary of risk of bias across domains.

**Figure 3:**
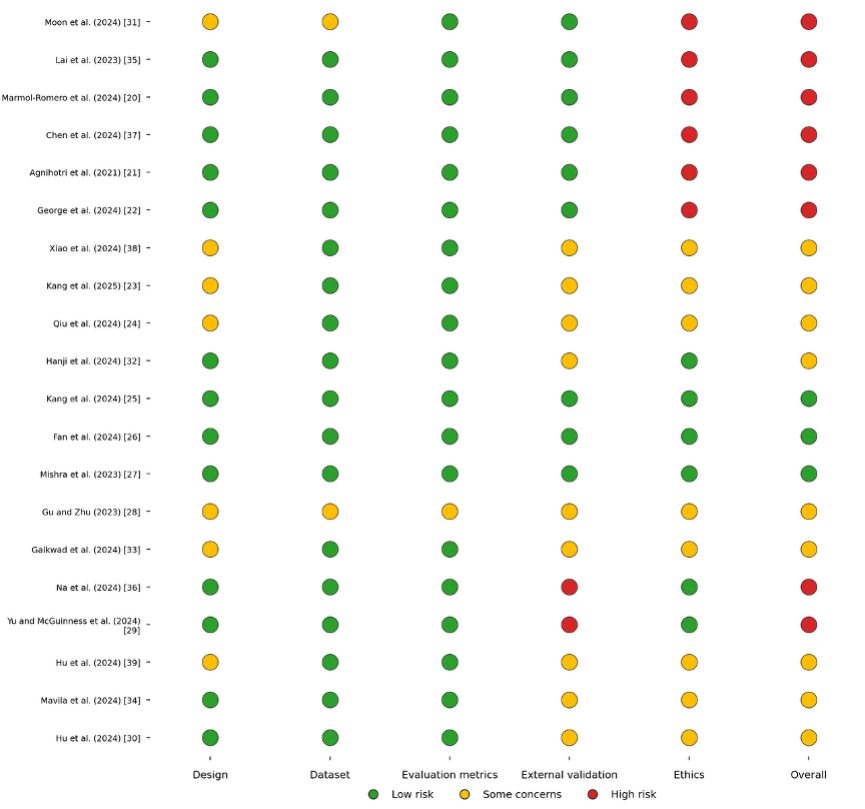
Traffic light plot for the assessment of risk of bias [20-39].

All screening, data extraction, and quality appraisal procedures were conducted in Covidence in accordance with PRISMA 2020 guidelines. Risk of bias was assessed using a structured traffic light framework covering 5 methodological domains: design, dataset reporting, evaluation metrics, external validation, and ethics, with an overall judgment summarized across domains. Detailed visualizations of study-level judgments are presented in Figures 2 and 3.

Two reviewers independently performed the assessments and resolved discrepancies (<5%) through discussion until consensus was reached. Domain-level ratings were assigned using study-reported methodological details, including eligibility criteria, dataset characteristics, predictor specification, outcome definitions, and validation strategies. Standardized Covidence extraction forms were used to support consistent evaluation across all included studies.

Across the design domain, most studies, such as Lai et al [35], Marmol-Romero et al [20], Chen et al [37], and George et al [22], were rated as low risk, with Moon et al [31], Gu and Zhu [28], Xiao et al [38], Kang and Hong [23], Qiu et al [24], Hu et al [39], and Gaikwad et al [33] resulting in some concerns (Figure 3) [20-39]. Evaluation metrics were similarly robust across the literature, with almost all studies receiving low-risk ratings, except Moon et al [31] and Gu and Zhu [28] showed some concerns. External validation represented the most prominent methodological limitation, with high risk identified for Yu and McGuinness [29] and Na [36]. Ethics-related reporting showed the greatest concentration of high-risk judgments, with Moon et al [31], Lai et al [35], Marmol-Romero et al [20], Chen et al [37], Agnihotri et al [21], and George et al [22] rated as high risk, while some concerns were observed for Xiao et al [38], Kang and Hong [23], Qiu et al [24], Gu and Zhu [28], Gaikwad et al [33], Hu et al [39], Mavila et al [34], and Hu et al [30]. Overall judgments reflected these patterns, indicating that while core reporting of datasets and evaluation metrics was generally adequate, gaps in external validation and ethics transparency remain key weaknesses in the current evidence base.

### Ethical Considerations

Ethical risks, including bias propagation, hallucinated outputs, and misinformation, were largely underexplored. Only 3 studies [28,31,35] briefly mentioned potential harms, and none systematically audited their models for safety in high-risk user scenarios. No study documented mitigation strategies for hallucinations or unintended outputs. Furthermore, training data provenance and annotation processes were rarely disclosed, making it difficult to assess content, privacy, or representational bias. Across the included studies, explicit ethical deployment frameworks, alignment with international AI governance guidelines (eg, World Health Organization [WHO] and Organisation for Economic Co-operation and Development [OECD]), or formal safety auditing protocols were rarely described, particularly in relation to vulnerable user populations.

## Results

### Study Characteristics

**Figure 4:**
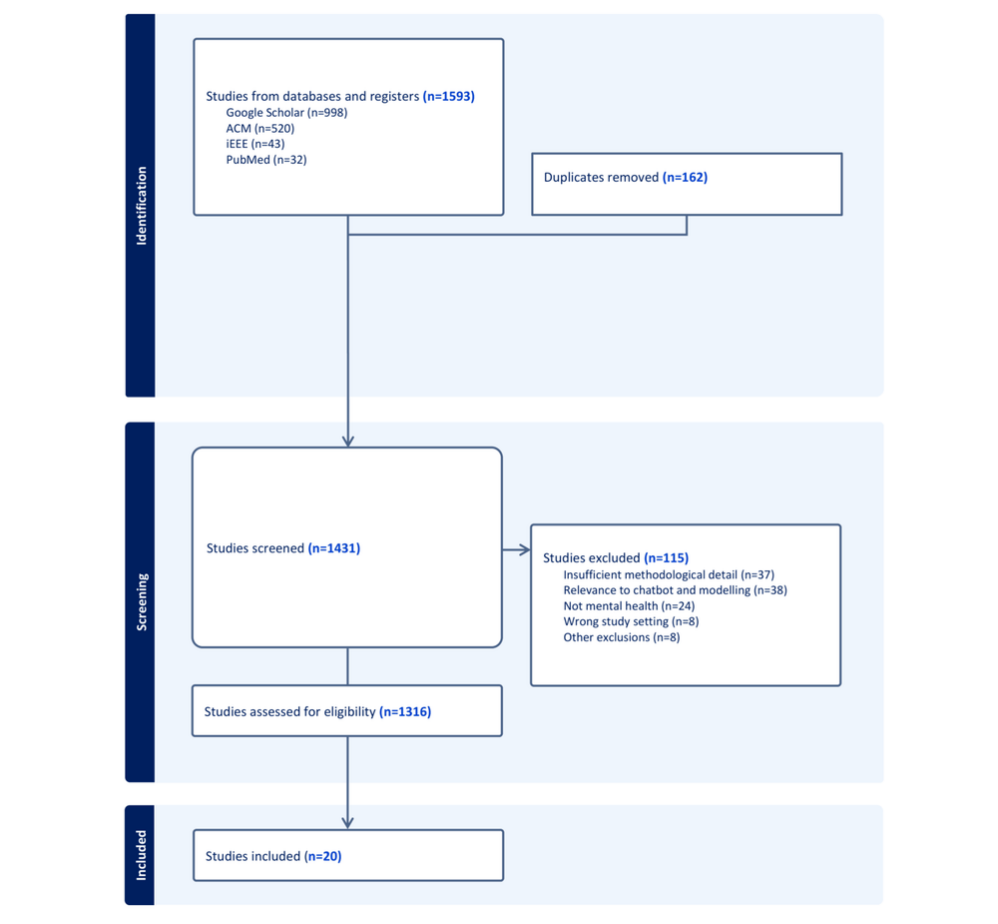
PRISMA 2020 flow diagram summarizing the identification, screening, and inclusion process for studies on large language model–based mental health counseling systems.

Our systematic review identified 20 studies [20-39] focusing on LLM-based MH chatbots and digital interventions (Figure 4). Notably, 95% (19/20) of the studies were published between 2023 and 2025, reflecting a rapid acceleration of research in this area. The distribution of publication venues varied, with 8 studies [1,4,7,9,12,15,18,20] appearing in MH-focused journals, 9 studies [2,3,5,6,8,10,11,14,17] in computer science conferences and journals, and 3 studies [13,16,19] in interdisciplinary domains such as medical informatics and AI applications in DMHI (Table 1). While some studies spanned multiple domains, each was counted once based on its primary publication type.

**Table 1:** Overview of LLM^a^-based mental health chatbots included in the review, summarizing study year, model name, underlying architecture, prompting strategy, and fine-tuning approach.

Author (year)	Model name	Integrated LLM	Prompt method	Fine-tuning
Moon et al [31] (2024)	—^b^	Mistral-7B Instruct-v0.2	Few-shot, CoT^c^, RAG^d^, and instruction tuning	Task-specific FT^e^ and FT-PEFT^f^ (QLoRA^g^)
Lai et al [35] (2023)	Psy-LLM	PanGu, Wen-Zhong	Few-shot, RAG, instruction tuning, and persona-based	Task-specific FT, FT-PEFT (LoRA^h^), instruction FT, and continual learning
Marmol-Romero [20] et al (2024)	—	GPT-3	Few-shot, prompt chaining, instruction tuning, and persona-based	—
Chen et al [37] (2024)	PBChat	ChatGLM2	Few-shot, CoT, prompt chaining, instruction tuning, and persona-based	Task-specific FT and FT-PEFT (QLoRA)
Agnihotri et al [21] (2021)	TACA	GPT-2	Few-shot, CoT, RAG, prompt chaining, instruction tuning, and persona-based	Task-specific FT, full FT, and instruction FT
George et al [22] (2024)	Mello	Mistral-7B Instruct v0.1	Few-shot, prompt chaining, instruction tuning, and persona-based	Task-specific FT, FT-PEFT (QLoRA), and instruction FT
Xiao et al [38] (2024)	HealMe	LLaMA2-7b-chat	Few-shot, CoT, prompt chaining, instruction tuning, and persona-based	Supervised fine-tuning and LoRA
Kang et al [23] (2025)	HoMemeTown Dr. CareSam	GPT-4.0	Few-shot, RAG, prompt chaining, instruction tuning, and persona-based	—
Qiu et al [24] (2024)	PsyChat	ChatGLM2-6B2	Few-shot, CoT, RAG, prompt chaining, instruction tuning, and persona-based	Task-specific FT, FT-PEFT (LoRA), and instruction FT
Hanji et al [32] (2024)	Self-Heal	GPT-2	Few-shot, prompt chaining, instruction tuning, persona-based, and multimodal input	Task-specific FT, full FT, and instruction FT
Kang et al [25] (2024)	—	LLaMA2-7B, ChatGLM2-6B	Few-shot, CoT, RAG, prompt chaining, and instruction tuning	Task-specific FT, FT-PEFT (inhibited LoRA), and RLHF^i^
Fan et al [26] (2025)	—	Qwen2-7B	Few-shot, CoT, RAG, prompt chaining, instruction tuning, and persona-based	Task-specific FT, FT-PEFT (LoRA), instruction FT (GPT-4 assisted), and RLHF
Mishra et al [27] (2023)	eTHERAPIST	GPT-2 Medium	Few-shot, prompt chaining, instruction tuning, and persona-based	Task-specific FT, full FT, instruction FT, and RLHF
Gu and Zhu [28] (2024)	MentalBlend	GPT-4, GPT-3.5-turbo	Few-shot, CoT, RAG, prompt chaining, instruction tuning, and persona-based	Task-specific FT
Gaikwad et al [33] (2024)	Sahara: Virtual Companion	DialoGPT + T5	Few-shot, RAG, prompt chaining, instruction tuning, persona-based, and multimodal input	Task-specific FT, full FT, and instruction FT
Na et al [36] (2024)	CBT-LLM	Baichuan-7B	Few-shot, CoT, RAG, prompt chaining, instruction tuning, and persona-based	Task-specific FT, FT-PEFT (LoRA), and instruction FT
Yu and McGuinness et al [29] (2024)	—	DialoGPT, ChatGPT 3.5	Few-shot, RAG, prompt chaining, instruction tuning, and persona-based	Task-specific FT and full FT
Hu et al [39] (2024)	—	Qwen-7B, Qwen-max	Few-shot, CoT, RAG, prompt chaining, instruction tuning, persona-based, and multimodal input	Task-specific FT, FT-PEFT (LoRA), and instruction FT
Mavila et al [34] (2024)	iCare	RASA, GPT-3	Few-shot, RAG, prompt chaining, instruction tuning, and persona-based	Task-specific FT and instruction FT
Hu et al [30] (2024)	PsycoLLM	Qwen1.5-14B-Chat	Few-shot, RAG, prompt chaining, instruction tuning, and persona-based	Full FT, task-specific FT, and instruction FT

^a^LLM: large language model.

^b^Not available.

^c^CoT: chain-of-thought.

^d^RAG: retrieval-augmented generation.

^e^FT: fine-tuning.

^f^PEFT: parameter-efficient fine-tuning.

^g^QLoRA: quantized low-rank adaptation.

^h^LoRA: low-rank adaptation.

^i^RLHF: reinforcement learning with human feedback.

### Model Overview

The reviewed studies varied in their application focus and deployment of LLM-based MH systems. Among the 20 studies [20-39], the majority (n=12) developed models aimed at general MH support, including conversational counseling, self-guided emotional regulation, and psychoeducational engagement. These included Marmol-Romero et al [20], TACA [21], Mello [22], HoMemeTown Dr. CareSam [23], PsyChat [24], Kang et al [25], Fan et al [26], e-THERAPIST [27], MentalBlend [28], Yu and McGuinness et al [29], PsycoLLM [30], and PBChat [37].

Six studies designed systems targeting specific psychological conditions such as depression, anxiety, or stress-related symptoms. For instance, Moon et al [31], Self-Heal [32], Sahara [33], iCare [34], Psy-LLM [35], and CBT-LLM [36] were explicitly grounded in condition-specific goals, with some aligned to structured therapeutic frameworks such as cognitive behavioral therapy (CBT). A smaller set of studies addressed niche applications, including behavioral pattern recognition (HealMe [38]) and child mental resilience (Hu et al [39]).

Notably, only 6 [21,22,24,31,32,38] out of 20 studies [20-39] provided open-source code or implementation details. The remaining 14 [20,23,25-30,33-37,39] studies did not release their models, limiting reproducibility and transparency. This pattern was associated with limited opportunities for independent benchmarking, external validation, and real-world adaptation of LLM-based digital MH tools.

### Dataset Sources and Preprocessing

The dataset sources and preprocessing methods varied significantly among the studies. A total of 13 studies used publicly available datasets, such as HOPE and MotiVAte Dataset (Moon et al [31]), ScenarioSA (TACA [21]), Counsel-Chat (Mello [22], iCare [34]), PATTERNREFRAME (HealMe [38]), SmileChat and Xingling (PsyChat [24]), Therapy_data (Self-Heal [32]), Alexander Street Therapy Data (Kang et al [23]), Emotional First Aid and Emotional-Support-Conversation Dataset (Fan et al [26]), PsyQA (Psy-LLM [35], CBT-LLM [36], MentalBlend [28]), and the Mental Health Corpus (Sahara: Virtual Companion [33]), enabling greater replicability. Six studies [20,25,27,29,30,39] relied on proprietary or unspecified datasets, including therapy session notes, real-world therapeutic conversations, and psychological counseling transcripts.

Text preprocessing was applied in 16 studies [20-24,26-36], primarily involving normalization, token cleanup, and content filtering to improve model robustness. Six studies [21,22,24,31,32,38] additionally used data augmentation techniques such as paraphrasing and backtranslation to enhance linguistic variability and generalizability. Tokenization methods varied as well: standard subword tokenization (eg, Byte Pair Encoding and WordPiece) was applied in 13 studies [20-22,24-26,28-33,37], while domain-specific tokenizers were implemented in 3 studies [33,35,36], particularly those working with non-English or clinical corpora.

### Model Architectures

The LLM architectures used across the 20 studies [20-39] reflected a range of general-purpose base models, instruction-tuned variants, and regionally adapted LLMs, rather than models inherently designed for MH applications. GPT-family models (GPT-2, GPT-3, GPT-3.5, and GPT-4) were the most commonly used, appearing in 9 studies [20-22,24,26,29-31,37], making them the dominant decoder-only generative architecture for counseling-oriented dialogue generation. These models were deployed in either zero-shot or fine-tuned configurations, depending on study objectives.

Beyond GPT, LLaMA-based models were used in 2 studies [28,35], and Mistral-based instruction-tuned models appeared in another 2 [28,35]. Importantly, Mistral itself is a general-purpose LLM and not intrinsically tailored to MH tasks; MH specificity in these studies was introduced through task-specific fine-tuning, prompt design, or alignment strategies rather than through the base architecture. Several studies also adopted region-specific LLMs for multilingual or non-English settings, including ChatGLM (3 studies [31,33,39]), Qwen (3 studies [25,34,38]), and Baichuan (1 study [26]), with applications often focused on Chinese-speaking populations. Additional generative models included DialoGPT and T5, which were selected for their conversational or encoder-decoder generation capabilities, while RASA was used primarily as a rule-based or hybrid dialogue management framework rather than a large generative model.

Importantly, no study used encoder-only architectures such as Bidirectional Encoder Representations from Transformers (BERT) as the core model for dialogue generation. Consistent with their design, encoder-based transformers are typically applied to classification, retrieval, or extractive question answering tasks, rather than open-ended response generation. The reviewed studies instead demonstrate a clear preference for decoder-only or encoder-decoder generative architectures that support few-shot prompting, instruction tuning, and flexible conversational output, which are better aligned with interactive MH counseling applications.

### Prompt Engineering

Prompt engineering was frequently reported as part of model design and evaluation across studies, though its independent impact on performance was not systematically quantified (Table 1). Few-shot prompting was used in 18 [20-37] of the 20 studies [20-39], providing models with minimal yet targeted examples to guide response generation. Instruction tuning, which aligns the model’s responses with therapist-like behavior or task-specific expectations, was found in 19 studies [20-33,35-39], reflecting its dominance in aligning LLM behavior with MH intervention goals.

Persona-based prompting was implemented in 18 studies [20-24,26-33,35-39] to simulate consistent, empathetic counselor personas. Prompt chaining, also used in 18 studies [20-29,31-33,35-39], enabled multistep reasoning and follow-up generation logic. Retrieval-augmented generation (RAG) was used in 15 studies [21-24,26-36] to incorporate external knowledge sources, such as clinical guidelines or internal documentation, into the response generation process. Chain-of-thought (CoT) prompting, designed to improve reasoning quality, was applied in 9 studies [22,24,26,29,31,33,35-37]. Only 3 studies [26,31,39] explored multimodal input prompting, indicating its nascent use in this space.

### Fine-Tuning Approaches

Fine-tuning strategies varied in scope and efficiency (Table 1). Task-specific fine-tuning was the most common, used in 18 studies [20-37] to tailor model behavior toward specific therapeutic objectives such as empathy generation, goal setting, or motivational interviewing. Instruction fine-tuning was reported in 12 studies [20-24,26,28-31,33,35], reinforcing alignment with counseling frameworks. Parameter-efficient fine-tuning (PEFT), including low-rank adaptation (LoRA) and its quantized variant quantized low-rank adaptation (QLoRA), was applied in 10 studies [21,22,24,26,28,30-32,35,38], often motivated by computational constraints or the need for modular updates.

Full fine-tuning, which requires more extensive retraining of the model, was performed in 6 studies [20,23,27,29,34,37]. Although computationally intensive, it enabled deeper customization. Reinforcement Learning from Human Feedback (RLHF) was implemented in 3 studies [31,33,36] to further refine model behavior based on human preferences and contextual alignment. Continual learning, the ability to incrementally update the model as new data become available, was mentioned in only one study [39], suggesting it remains an underexplored area in LLM-based MH systems.

### Evaluation Metrics

Evaluation methods across the included studies spanned both qualitative and quantitative approaches (Table 2). Human evaluation was reported in 18 [20-37] of the 20 studies [20-39], with assessments commonly carried out by MH professionals, trained researchers, or domain experts. These evaluations focused on response quality, contextual relevance, empathetic tone, and overall helpfulness. Five studies went further by evaluating subjective dimensions such as perceived trust, safety, and user comfort, reinforcing the complexity and sensitivity of human-chatbot interactions in MH contexts.

A total of 18 studies [20-31,33-38] incorporated quantitative evaluation methods, reflecting growing efforts to standardize outcome measurement. Among these, lexical overlap metrics were the most frequently reported: BLEU, ROUGE, and distinct-n scores were used in 11 studies [24,25,27-31,33,35-37] to assess textual similarity or diversity between model-generated responses and reference utterances. These metrics, while commonly applied in natural language generation, provide limited insight into conversational appropriateness or therapeutic value. Six studies [24,27,29,33,35,36] used fluency and coherence metrics, including perplexity and linguistic entropy, to quantify the structural quality and readability of generated responses. Despite the emphasis on linguistic metrics, only a small subset used validated psychometric instruments, such as depression screening scales or evidence-based dialogue frameworks. This indicates a significant gap in the clinical grounding of current evaluation pipelines. No studies reported the use of instruments such as the Patient Health Questionnaire-9 (PHQ-9), Generalized Anxiety Disorder-7 (GAD-7), or System Usability Scale (SUS) in a standardized clinical setting, and no study reported results from a registered or randomized controlled trial.

**Table 2:** Evaluation metrics used across included studies, distinguishing between automated NLP^a^-based measures and human-assessed qualitative metrics.

Author (year)	Evaluation metrics (automatic)	Evaluation metrics (human)	Human evaluation (N)
Moon et al [31] (2024)	*F*_1_-score, BLEU^b^, ROUGE-L^c^, and BERTScore^d^	Human/Expert (activation event, beliefs, consequences, and distorted parts)	Annotators (n=2)
Lai et al [35] (2023)	Perplexity, ROUGE-L, and distinct-n metrics	Human/Expert (helpfulness, fluency, relevance, and logic)	Human evaluators (n=6)
Marmol-Romero et al [20] (2024)	Frequency and count of messages per session and gamification participation	Human/Expert (satisfaction, frequency of use, meeting expectations, problems encountered, preferences, and motivations)	Not reported
Chen et al [37] (2024)	BLEU (BLEU-1, BLEU-2, BLEU-3, BLEU-4), ROUGE^e^ (ROUGE-1, ROUGE-2, ROUGE-L)**,** BERTScore	Human/Expert (consistency, pertinency, rationality, accuracy, and relevancy)	Human evaluation sample size (n=409)
Agnihotri et al [21] (2021)	Accuracy and *F*_1_-score	Human/Expert (emotional relevance and contextual relevance)	Not reported
George et al [22] (2024)	PsychoBench: Emotional Intelligence Scale and Empathy Scale	No human evaluation	Not reported
Xiao et al [38] (2024)	GPT-4 was used to automatically rate the AI^f^ responses in empathy, logical coherence, guidance, and overall score	Human/Expert (empathy, coherence, and guidance), User (PANAS [Positive and Negative Affect Schedule] before and after therapy)	Human evaluation (n=8)
Kang et al [23] (2025)	NLP-based analysis against *DSM-5*^g^ criteria	Human/Expert (interviews and open-ended survey), Users Rating (empathy, accuracy, usefulness, complex thinking, emotions, active listening, appropriate questions, positivity, support, professionalism, and personalization)	Participants (n=20)
Qiu et al [24] (2024)	Accuracy, *F*_1_-score, perplexity, METEOR^h^, BLEU-1/2/3, ROUGE-L, and distinct-1/2	Human/Expert and user surveys (fluency, coherence, empathy, usefulness, and appropriateness)	Not reported
Hanji et al [32] (2024)	—^i^	Human/Expert (empathy, coherence, sentiment adaptation, and user engagement quality)	Not reported
Kang et al [25] (2024)	ROUGE and fluency	Human/Expert (readability, professionalism, and match score)	Not reported
Fan et al [26] (2025)	Accuracy, precision, recall, GPT-4 to evaluate (semantic similarity, fluency, expertise, and empathy)	Human/Expert: professionalism, fluency, and empathy	Human-related samples (n=200)
Mishra et al [27] (2023)	Accuracy (W-ACC^j^) and macro-*F*_1_, perplexity**,** BERTScore-*F*_1_, and response length	Human/Expert: consistency/correctness (gender-age, persona, psychotherapeutic approach, politeness, interpersonal behavior), fluency, consistency, and nonrepetitiveness	Human experts (n=6) and annotators (n=3)
Gu and Zhu [28] (2024)	BLEU score (1, 2, 3, 4, avg), BERTScore (PBERT, RBERT, and FBERT), distinct-2	Human/Expert (fluency, helpfulness, relevance, empathy, and professionalism)	Human experts (n=6)
Gaikwad et al [33] (2024)	Word error rate, accuracy, and perplexity	No human evaluation	Not reported
Na et al [36] (2024)	Accuracy, recall, *F*_1_-score, BLEU, METEOR, CHRF^k^, BLEURT^l^, BERTScore	Human/Expert (Relevance, CBT structure, and helpfulness)	Human evaluators (n=6)
Yu and McGuinness et al [29] (2024)	Perplexity and BLEU	User/Professional evaluation (perceived utility, usage willingness, human-likeness, supportiveness, and overall rating)	Participants (n=20; 10 patients, 10 professionals)
Hu et al [39] (2024)	—	User satisfaction, qualitative feedback on authenticity, entertainment value, fluency, and usefulness	Participants (n=48) + Interviews (n=10)
Mavila et al [34] (2024)	Accuracy, precision, recall, and *F*_1_-score	User satisfaction (empathy, relevance, and helpfulness)	Not reported
Hu et al [30] (2024)	Standard, elastic accuracy, ROUGE-1 and ROUGE-L, BLEU-4, BERTScore	Human/Expert (assess and eliminate low-quality data)	Not reported

^a^NLP: natural language processing.

^b^BLEU: Bilingual Evaluation Understudy.

^c^ROUGE-L: Recall-Oriented Understudy for Gisting Evaluation Longest Common Subsequence.

^d^BERTScore: Bidirectional Encoder Representations from Transformers Score.

^e^ROUGE: Recall-Oriented Understudy for Gisting Evaluation.

^f^AI: artificial intelligence.

gDSM-5: Diagnostic and Statistical Manual of Mental Disorders (Fifth Edition).

^h^METEOR: Metric for Evaluation of Translation with Explicit Ordering.

^i^Not available.

^j^W-ACC: weighted accuracy.

^k^CHRF: character n-gram F-score.

^l^BLEURT: BLEU with Representations from Transformers.

### Performance Outcomes

Reported outcomes differed across studies using different architectures, fine-tuning strategies, and evaluation approaches; however, direct performance comparisons were not feasible due to heterogeneity in study design and metrics. Studies that applied task-specific or instruction-based fine-tuning consistently reported stronger outcomes across both human and automated assessments. These models demonstrated higher topic coherence, contextual appropriateness, and emotional responsiveness. For instance, instruction-tuned models were often evaluated using human-rated empathy and relevance criteria, with several studies reporting higher scores relative to their own baselines, zero-shot GPT variants.

Conversely, models evaluated under zero-shot conditions or deployed without domain adaptation tended to struggle with emotionally sensitive content. These models often produced generic or inconsistent responses, with several studies reporting lower scores on fluency, helpfulness, or therapeutic fit. Some models failed to maintain coherent dialogue across multiple turns, especially when faced with ambiguous or distress-laden prompts.

Qualitative findings reinforced these gaps. While several studies noted strengths in fluency, turn-taking, and user engagement, others identified persistent issues such as repetitiveness, surface-level advice, and lack of empathy. User trust and perceived empathy were reported inconsistently; some users described feeling supported, while others perceived the chatbot as impersonal or emotionally disconnected. A few studies highlighted the trade-off between linguistic quality and therapeutic value. Models that generated fluent and natural-sounding text often lacked deeper reflective or validating responses expected in clinical contexts. Only a small subset of studies used hybrid evaluation frameworks that integrated human rubrics (eg, CARE [Consultation and Relational Empathy], supportiveness, and validation) with NLP metrics, suggesting a need for more comprehensive, clinically informed evaluation pipelines.

Performance outcomes across the studies showed notable variation depending on model type, evaluation strategy, and adaptation method. For example, the e-THERAPIST model [27] achieved high automatic evaluation scores, including a gender-age consistency of 90.1%, persona consistency of 84.1%, and psychotherapeutic approach correctness of 92.6%. Its human evaluation scores were similarly high, with fluency rated at 4.62 and consistency at 4.60. The HealMe model [38], designed to identify negative thought patterns, reported a perplexity of 2.52 and a BERTScore (Bidirectional Encoder Representations from Transformers Score)-*F*_1_ of 0.89, indicating both lexical and contextual fluency. In comparative benchmarking, PBChat [37] outperformed baseline ChatGLM2 across all major metrics, with ROUGE-L (Recall-Oriented Understudy for Gisting Evaluation Longest Common Subsequence) scores of 28.18 (PanGu) and 23.56 (WenZhong), and distinct-n scores up to 12.74. Helpfulness and fluency in PBChat were rated 3.87 and 4.36, respectively, based on expert review. Meanwhile, models relying solely on zero-shot inference (eg, standard GPT without fine-tuning) tended to underperform, with lower human-rated empathy and coherence, especially in complex or emotionally sensitive scenarios. Despite strong performance in specific metrics, only 3 studies [22,23,38] used psychometrically grounded tools (eg, PHQ-9) to evaluate MH alignment, indicating a persistent gap in clinically validated performance benchmarking.

### Service Approach

The reviewed studies explored diverse service models for deploying LLMs. The most common approach was the deployment of standalone chatbot systems, reported in 18 studies [20-37], often via web-based platforms offering real-time conversational support. Prominent examples of standalone deployments include Psy-LLM [35], PBChat [37], TACA [21], Mello [22], HealMe [38], PsyChat [24], MentalBlend [28], Sahara: Virtual Companion [33], CBT-LLM [36], and e-THERAPIST [27]. These systems were typically accessed through browser interfaces or dedicated portals, offering direct user-chatbot interaction without integration into broader ecosystems.

Virtual agents were used in 4 studies (PsyChat [24], Kang et al [25], and PsycoLLM [30], Moon et al [31]), where LLMs were embedded as AI-driven entities within broader service frameworks. These agents were designed to simulate therapist-like roles or guide users through decision support, often involving role-based or persona-enhanced interaction strategies beyond traditional rule-based chatbot systems. Only 2 studies [20,29] implemented embedded chatbot functionality within existing platforms. Yu et al [29] integrated their chatbot into a Unity-based MH simulation (Unity Technologies), while Marmol-Romero et al [20] deployed a Telegram-based chatbot interface (Telegram Messenger LLP) to improve user reach and reduce barriers to entry.

Notably, only one study (Self-Heal [32]) explicitly reported building a mobile app, suggesting limited attention to app-based delivery despite mobile devices being a primary access point for DMHI globally. Importantly, no study used a hybrid deployment strategy (eg, simultaneous deployment via web, mobile, and messaging platforms), highlighting a gap in cross-platform interoperability and multimodal user engagement design. Most of the current implementations remain single-channel, limiting their potential reach and integration into broader digital health ecosystems.

### Risk of Bias and Study Quality

Using our structured traffic light quality appraisal across 5 methodological domains (design, dataset reporting, evaluation metrics, external validation, and ethics), most studies demonstrated low risk for dataset reporting and evaluation metrics, suggesting that data sources and performance measures were generally well described (Figures 2 and 3). Design quality was also predominantly rated as low risk, with only a small subset of studies showing some concerns. In contrast, external validation emerged as the most frequent limitation, with several studies rated as some concerns and a few rated as high risk due to limited independent validation or incomplete reporting of validation procedures. Ethics-related reporting showed the greatest variability, with multiple studies rated as high risk and others rated as some concerns, reflecting inconsistent reporting of privacy safeguards, governance oversight, and safety considerations.

### Transparency and Reproducibility

Reproducibility remains a significant limitation in LLM-based MH chatbot research. Among the 20 included studies [20-39], only 6 [21,22,24,31,32,38] provided public access to source code or pretrained models, and 4 [21,22,31,38] shared any portion of their datasets. Most studies relied on proprietary or institution-specific data without sufficient documentation to support independent reimplementation. Beyond the limited availability of source code, few studies provided sufficient documentation of preprocessing pipelines, prompt templates, or evaluation protocols to enable full reproducibility. This lack of end-to-end transparency was associated with limited opportunities for independent benchmarking, external validation, and assessment of real-world applicability.

### Geographic and Cultural Context

Several studies used region-specific language models and datasets, reflecting diverse linguistic and cultural contexts. For example, ChatGLM and Qwen models were used in Chinese-language applications, while models such as Baichuan and PanGu were also designed for East Asian populations. Despite this, few studies explicitly discussed cultural adaptation strategies or localized evaluation. No study addressed cross-cultural generalizability or model fairness across ethnic or regional groups. Given the sociocultural nuances of MH, studies should emphasize linguistic inclusivity, cultural sensitivity, and population-specific validation in LLM-based applications.

### Cross-Study Synthesis

Cross-study synthesis revealed 3 recurring patterns across the included studies. First, evaluation approaches predominantly relied on linguistic or proxy performance metrics, such as automated text similarity scores or rubric-based qualitative ratings, whereas clinically grounded or psychometric outcome measures were infrequently reported. Only a small subset of studies incorporated validated MH instruments, and these were typically applied in research-specific contexts rather than standardized clinical workflows. Second, validation practices were limited across the literature. Few studies conducted external validation using independent datasets or user populations, and reporting of rater calibration, interrater reliability, or structured evaluation protocols was uncommon. Most evaluations were conducted within single-study settings, often using bespoke or internally defined assessment criteria, which constrained comparability across studies. Third, the reporting of safety mechanisms and governance strategies varied substantially. While some studies described the use of prompt constraints, refusal strategies, or rule-based safeguards to mitigate harmful outputs, many provided limited detail on how safety risks were operationally addressed or monitored. Formal descriptions of escalation pathways, bias auditing procedures, or governance frameworks were inconsistently reported across the included studies.

### Cost and Resource Reporting

Computational efficiency was rarely described across the included studies. Key deployment details, such as parameter count, hardware specifications, training duration, and inference latency, were often omitted. Only a small subset mentioned resource-saving adaptations, most commonly parameter-efficient fine-tuning. Limited reporting on compute and runtime characteristics restricted the assessment of feasibility and real-world readiness.

### Data Availability and Reporting

Not all studies provided complete information for every evaluation criterion. Some studies lacked details on specific aspects such as dataset sources, fine-tuning strategies, tokenization methods, or evaluation metrics. In these cases, the corresponding fields were marked as NA in our analysis. As a result, the total study counts for individual categories did not always sum to 20 (Tables 1 and 2). This reflects variability in reporting standards across studies and highlights the need for more standardized methodologies in LLM-based MH chatbot evaluations.

### Emerging Trends and Innovation

Recent trends reveal a shift from static, single-turn chatbots to dynamic multiagent conversational systems and multimodal AI companions. Several studies began incorporating RAG, CoT prompting, and instruction tuning to simulate reasoning and improve empathy. Others explored modular architectures and hybrid agent systems capable of collaborative role-play, such as therapist-patient-coach simulations. Although still in early stages, these advances highlight the growing complexity and potential of next-generation LLM-based DMHIs.

## Discussion

### Principal Findings

Our review highlights the increasing adoption of LLM-based models in MH counseling, with a predominant reliance on GPT-based architectures. Despite improvements in natural language understanding and conversational adaptability, several limitations persist in current implementations. The lack of clinical benchmarking raises concerns about the real-world applicability and safety of these models. The overwhelming use of proprietary datasets restricts reproducibility, making it difficult to generalize findings across diverse populations. Furthermore, evaluation frameworks remain inconsistent, with studies relying on general NLP performance metrics rather than clinically meaningful outcome measures. While fine-tuning strategies were used in several studies, their impact on response accuracy and clinical relevance remains underexplored. Fine-tuned models demonstrated better alignment with MH-specific dialogues, but the trade-offs in computational cost and dataset limitations restricted their scalability. The effectiveness of prompting techniques also varied, with few-shot prompting outperforming zero-shot prompting in maintaining contextual awareness and improving response coherence. However, CoT prompting, which could enhance multistep reasoning in counseling interactions, was rarely used.

### Study Limitations

Several methodological limitations should be acknowledged. First, the search was limited to English-language publications, which may have excluded relevant non-English work. Second, the relatively small number of eligible studies and their methodological heterogeneity constrained the generalizability of our findings. Third, due to variations in modeling approaches and outcome reporting, a formal meta-analysis could not be performed, and the synthesis remains descriptive rather than quantitative. Finally, interstudy variability in reporting model architecture and evaluation metrics constrained comparability across results. Future systematic reviews could address these issues by expanding database coverage, including multilingual evidence, and conducting meta-analytic modeling once more homogeneous data become available.

### Regulatory Readiness and Clinical Integration

From a counseling practice perspective, the predominance of technical performance metrics over clinically grounded outcomes limits the interpretability of reported effectiveness for real-world MH care. Without standardized psychometric evaluation, external validation, or integration into established care pathways, current LLM-based counseling systems remain difficult to assess in terms of therapeutic appropriateness, risk management, and clinical utility. These limitations may hinder clinician trust and slow responsible integration into digital MH service delivery.

None of the reviewed studies reported formal clinical validation, trial registration, or engagement with regulatory bodies such as the Food and Drug Administration (FDA). While a few referenced potential deployment in counseling or therapy contexts, no study implemented the chatbot in a controlled clinical workflow or evaluated clinical efficacy using standard instruments. Integration into digital MH care systems therefore remains conceptual. Bridging this gap will require interdisciplinary collaboration with clinicians, formal usability testing, and alignment with medical device regulations and health data standards.

Although ethical risks such as bias, hallucinated outputs, and inappropriate reassurance were frequently acknowledged, few studies provided operational detail on how these risks were monitored or mitigated. Formal mechanisms for handling high-risk disclosures, escalation to human professionals, or postdeployment monitoring were rarely described. Moreover, explicit engagement with regulatory pathways, clinical oversight models, or medical device governance frameworks was largely absent. These gaps raise concerns regarding regulatory readiness and highlight the need for stronger ethical and clinical infrastructure prior to large-scale deployment in MH settings.

### Cost and Resource Efficiency

Most studies did not report details on model size, inference latency, or computational requirements. Only a few mentioned using parameter-efficient fine-tuning techniques such as LoRA to reduce training cost. This absence of cost-awareness limits the practical deployability of many proposed models, particularly in resource-constrained operational environments. In clinical settings, hospitals and MH clinics, infrastructure budgets are often tightly constrained and must balance multiple priorities. High resource demands may hinder scalability or lead to reliance on external cloud services, which raises further concerns about data privacy, latency, and long-term operational cost. Future work should explicitly report hardware constraints, training time, model size, and inference cost for potential use in low- and middle-income countries or high-demand clinical settings where efficient and secure solutions are essential.

### Comparison With Prior Work

While prior systematic reviews have explored various aspects of LLM-driven MH applications, crucial gaps remain in assessing their real-world utility and clinical effectiveness. Studies such as Guo et al [10] and Hua et al [9] examined LLM applicability in MH but provided limited assessments of conversational interventions. Other reviews focused on safety [40] and ethical concerns [41,42], yet lacked discussions on practical deployment, user adoption, and chatbot-specific challenges remain scarce. Additionally, existing studies lack standardized evaluation frameworks for assessing MH chatbot interactions, with few exceptions, such as Abbasian et al [43], proposing structured metrics that remain largely unvalidated in clinical settings. Despite the increasing adoption of LLMs in DMHI, there is no systematic comparison of fine-tuning techniques, prompt engineering, or model-specific adaptations for MH chatbots.

Compared to earlier literature that often treated LLMs as monolithic tools or black-box models, recent studies [44,45] have begun refining their roles with DMHI through structured prompt design, domain adaptations, explainable LLM, and human-in-the-loop feedback. However, technological advances continue to emerge rapidly, while a gap between experimental prototypes and deployable, interpretable, patient-safe tools persists. This systematic review contributes to the field by systematically analyzing 20 LLM-based MH chatbots across deployment models, prompting strategies, and evaluation designs, assessing their real-world adoption and accessibility.

### Limitations and Directions for Future Modeling

A key limitation across current LLM-based MH chatbots is limited trustworthiness and interpretability. Many systems rely on black-box generative models without incorporating explicit explainability mechanisms, raising concerns regarding user confidence and the safety of automated MH support. Although most models enable real-time conversational interaction, their adaptive capabilities remain constrained. Rather than dynamically responding to evolving user context, most systems operate through predefined conversational structures. Personalization is similarly limited, as few models incorporate user history, sentiment signals, or behavioral context to inform tailored responses. A small number of studies suggest the potential value of more adaptive or multimodal designs; however, existing evidence remains preliminary and insufficient to establish their effectiveness or clinical viability.

Despite growing interest in evaluation, substantial gaps in performance reporting continue to limit cross-study comparison and generalizability. While several studies referenced user satisfaction, most reported it as a binary outcome without quantifying engagement depth or therapeutic relevance. Although the SUS was mentioned in a small number of studies, none reported numeric scores, constraining benchmarking of usability and acceptability. Expert evaluation frameworks were inconsistently described, with limited reporting on rating dimensions, scale definitions, or interrater reliability. Response time, a critical usability factor, was occasionally noted but not systematically measured. Some studies introduced internally defined scoring schemes, such as the Emotional Impact Score (EIS), but these lacked standardized definitions. Collectively, these inconsistencies underscore the need for more structured, transparent, and clinically grounded evaluation pipelines.

In addition, most studies did not disclose detailed information regarding training data provenance or fine-tuning procedures, limiting transparency and reproducibility. While several works explored increasingly complex system designs, formal safeguards related to ethical governance, secure data handling, and clinical oversight were rarely specified. Advancing toward clinically deployable LLM-based MH systems will require a rigorous external validation framework, standardized outcome measurement, and explicit attention to regulatory and ethical alignment.

Taken together, current evidence indicates that LLM-based counseling systems remain at an early developmental stage characterized by rapid technical experimentation but limited empirical grounding. Progress in this domain will depend on the adoption of standardized evaluation frameworks that integrate both NLP-based and psychometric measures, transparent reporting of model development and validation practices, and multidisciplinary collaboration among clinicians, data scientists, and ethicists to support safe, equitable, and clinically accountable deployment.

### Conclusions

This systematic review highlights the growing use of LLM-based chatbots in MH counseling. While these models show promise in enhancing accessibility and response adaptability, significant challenges remain, including a lack of clinical validation, ethical safeguards, and standardized evaluation frameworks. Addressing these gaps will be critical in transitioning LLM-based chatbots from experimental tools to clinically trusted interventions.

## Appendix

Multimedia Appendix 1 Search queries.

Multimedia Appendix 2 Glossary of key terms.

Multimedia Appendix 3 PRISMA checklist.

## Abbreviations

AI: artificial intelligence
BERT: Bidirectional Encoder Representations from Transformers
BERTScore: Bidirectional Encoder Representations from Transformers Score
BLEU: Bilingual Evaluation Understudy
CARE: Consultation and Relational Empathy
CBT: cognitive behavioral therapy
CoT: chain-of-thought
DMHI: digital mental health intervention
EIS: Emotional Impact Score
FDA: Food and Drug Administration
GAD-7: Generalized Anxiety Disorder-7
LLM: large language model
LoRA: low-rank adaptation
MH: mental health
NLP: natural language processing
OECD: Organisation for Economic Cooperation and Development
PEFT: parameter-efficient fine-tuning
PHQ-9: Patient Health Questionnaire-9
PRISMA: Preferred Reporting Items for Systematic Reviews and Meta-Analyses
QLoRA: quantized low-rank adaptation
RAG: retrieval-augmented generation
RLHF: reinforcement learning with human feedback
ROUGE: Recall-Oriented Understudy for Gisting Evaluation
ROUGE-L: Recall-Oriented Understudy for Gisting Evaluation Longest Common Subsequence
SUS: System Usability Scale
WHO: World Health Organization
