# Supplementary material for: Large Language Model–Based Chatbots and Agentic AI for Mental Health Counseling: Systematic Review of Methodologies, Evaluation Frameworks, and Ethical Safeguards
Source: JMIR AI. 2026 Mar 13;5:e80348. doi: 10.2196/80348 (PMC13032092; doi:10.2196/80348)
Supplement: Multimedia Appendix 1 [file ai_v5i1e80348_app1.docx]

**Multimedia Appendix 1.** Search Queries.

PubMed:

("large language model" OR "LLM" OR "LLMs")

AND

("mental health" OR "mental illness" OR "mental disorder" OR "mental wellness")

AND

("chatbot" OR "counseling" OR "conversational agent" OR "digital intervention" OR "DMHI")

IEEE Xplore:

("large language model" OR "LLM")

AND

("mental health" OR "mental disorder")

AND

("chatbot" OR "counseling" OR "conversational")

ACM Digital Library:

("large language model" OR "LLM")

AND

("mental health")

AND

("chatbot" OR "conversational agent")

Google Scholar:

"large language model" AND "mental health" AND (chatbot OR counseling)
