# Supplementary material for: Large Language Model–Based Chatbots and Agentic AI for Mental Health Counseling: Systematic Review of Methodologies, Evaluation Frameworks, and Ethical Safeguards
Source: JMIR AI. 2026 Mar 13;5:e80348. doi: 10.2196/80348 (PMC13032092; doi:10.2196/80348)
Supplement: Multimedia Appendix 2 [file ai_v5i1e80348_app2.docx]

**Multimedia Appendix 2.** Glossary of Key Terms.

The following glossary provides operational definitions of key technical and methodological terms as used in this systematic review. Definitions are usage-based and reflect how terms are applied across the included studies.

Agentic AI: LLM-driven systems capable of goal-directed or role-conditioned behavior across multi-step interactions, often involving adaptive decision-making or coordinated conversational roles.

LLM-Based Mental Health Chatbots: Conversational systems powered by large language models that generate natural language responses to provide mental health-related support, guidance, or counseling-oriented dialogue.

Rule-Based Chatbots: Conversational systems that rely on predefined rules, decision trees, or scripted responses, without generative language modeling or contextual learning capabilities.

Virtual LLM Agent: An LLM-powered conversational entity designed to simulate a specific role (e.g., therapist, coach, guide) within an interactive system, often embedded within a broader service framework.

Persona-Based Prompting: A prompting strategy that conditions an LLM to adopt a consistent role, identity, or counseling style throughout an interaction.

Prompt Chaining: A structured prompting technique in which multiple prompts are sequentially applied to guide multi-step reasoning, reflection, or response refinement.

Multimodal Input Prompting: An interaction design in which non-textual inputs (e.g., images, audio, physiological signals) are incorporated alongside text to inform response generation.

Single-Turn Chatbots: Conversational systems that generate responses independently for each user input without maintaining conversational history or contextual memory across turns.

Modular Architectures: System designs composed of multiple interacting components (e.g., dialogue manager, safety filter, retrieval module) rather than a single monolithic model.

Hybrid Agent Systems: Systems that combine multiple interaction paradigms, such as LLM-based generation with rule-based control, retrieval modules, or human-in-the-loop oversight.

Broader Service Frameworks: Digital environments in which chatbots or agents are embedded as one component of a larger mental health service ecosystem (e.g., platforms integrating assessment, monitoring, or referral).

Standardized Clinical Setting: A controlled healthcare or research environment in which interventions are delivered according to established clinical protocols, often involving validated instruments, clinician oversight, or trial registration.

Key Counseling Metrics: Evaluation dimensions used to assess counseling-oriented interactions, such as empathy, relevance, helpfulness, appropriateness, and therapeutic alignment, typically measured through human or rubric-based evaluation.

Unity-Based Deployment: Implementation of a chatbot or agent within a Unity-powered simulation or interactive environment, often used for embodied or scenario-based mental health applications.

Telegram-Based Deployment: Delivery of chatbot functionality through the Telegram messaging platform to facilitate accessibility and user engagement via existing communication channels.
